# Biomechanical Considerations in the Design of High-Flexion Total Knee Replacements

**DOI:** 10.1155/2014/205375

**Published:** 2014-05-06

**Authors:** Cheng-Kung Cheng, Colin J. McClean, Yu-Shu Lai, Wen-Chuan Chen, Chang-Hung Huang, Kun-Jhih Lin, Chia-Ming Chang

**Affiliations:** ^1^Institute of Biomedical Engineering, National Yang-Ming University, No. 155, Section 2, Linong Street, Beitou District, Taipei 11221, Taiwan; ^2^Orthopaedic Device Research Center, National Yang-Ming University, No. 155, Section 2, Linong Street, Beitou District, Taipei 11221, Taiwan; ^3^Department of Medical Research, Mackay Memorial Hospital, New Taipei City 10499, Taiwan; ^4^Center of Translation Technology for Medical Device, Chung Yuan Christian University, Taoyuan 32023, Taiwan

## Abstract

Typically, joint arthroplasty is performed to relieve pain and improve functionality in a diseased or damaged joint. Total knee arthroplasty (TKA) involves replacing the entire knee joint, both femoral and tibial surfaces, with anatomically shaped artificial components in the hope of regaining normal joint function and permitting a full range of knee flexion. In spite of the design of the prosthesis itself, the degree of flexion attainable following TKA depends on a variety of factors, such as the joint's preoperative condition/flexion, muscle strength, and surgical technique. High-flexion knee prostheses have been developed to accommodate movements that require greater flexion than typically achievable with conventional TKA; such high flexion is especially prevalent in Asian cultures. Recently, computational techniques have been widely used for evaluating the functionality of knee prostheses and for improving biomechanical performance. To offer a better understanding of the development and evaluation techniques currently available, this paper aims to review some of the latest trends in the simulation of high-flexion knee prostheses.

## 1. Introduction

Many daily activities require considerable knee flexion, level walking >60°, ascending stairs >80°, sitting >90°, and getting out of a bath >130° [1, 2]. High-flexion often refers to movements that require over 120° of knee flexion, which are particularly common in Asian cultures [3–6], squatting, sitting cross-legged, kneeling, and prayer. High-flexion (HF) knee prostheses have been developed for this purpose and have been proven to accommodate such movements. However, whether such HF prostheses are clinically more effective than conventional knee replacements is debatable, with most studies showing either no significant improvement or mild improvements over conventional TKA [7–10]. Even for studies that do report significantly greater flexion with HF designs, bias in patient selection, experimental errors, or shortcomings in measurement methods may greatly influence the results [3–5]. Therefore, whether HF knee prostheses are practically useful to patients requires further research using clearly defined measurement and testing methods to make studies comparable.

The choice to use a high-flexion knee is ultimately left to the surgeon, with patient consent. As such, this report will compile published data regarding the biomechanical aspects of HF total knee replacement (TKR) with special focus on posterior cruciate ligament retaining knees (cruciate-retaining (CR)) and posterior cruciate ligament sacrificing designs (posterior-stabilized (PS)). Meta-analyses comparing CR and PS knees have generally shown no favourable design in terms of longevity, range of motion, and pain. A recent Cochrane Database review [11] of 17 studies involving 1810 patients concluded that there is no solid clinical reason for choosing to either retain or remove the posterior cruciate ligament (PCL) but also noted that arthroplasty where the PCL is retained is more difficult to perform.

## 2. Modelling High Knee Flexion

This review details two computational methods for studying the biomechanics of knee prostheses. One method uses multibody dynamics software, namely, MSC Adams (MSC Software Corporation, Santa Ana, CA), to study the dynamic behaviour of the knee joint. Another technique is to use finite element analysis (FEA) to study the internal mechanical condition of the joint: stress, strain, and so forth. The FEA research detailed below typically used ABAQUS (Dassault Systèmes, Vélizy-Villacoublay, France) accompanied by some preprocessing and meshing software.

The major drawback in the computational simulation of joints lies in the simplifications that must be made within each model. Some of these assumptions are purposely designed into the model in consideration of cost, computing power, time, processing, and so forth, but it must also be noted that such simulations have not advanced far enough to realistically imitate all of the components and tissues within the joint. Inherent simplifications in the modelling process, such as inaccurate representations of soft tissues, need to be taken into consideration. The validity of a model may be determined by comparing the results against in/ex vivo studies of a representative human joint or by comparing the models against previously validated simulations. Each of the models detailed below has been validated in this manner.

## 3. Achieving High Knee Flexion

**Figure 1:**
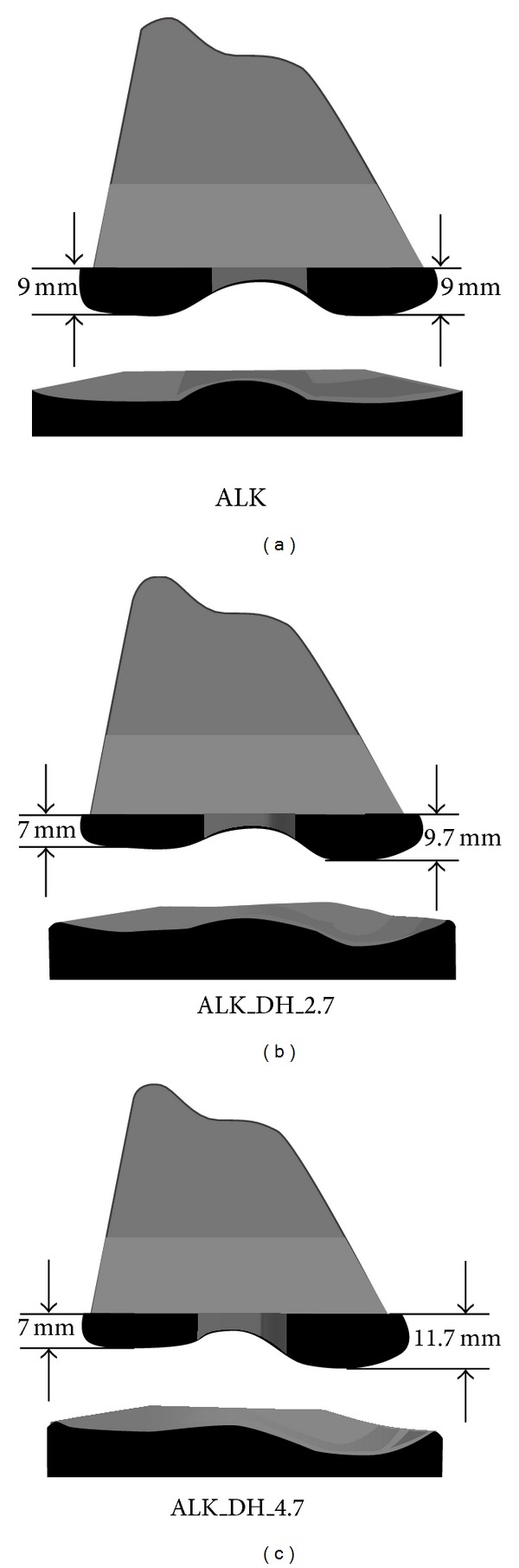
Femoral and tibial components modified on both medial and lateral sides [18]. (a) Anatomic-like knee, (b) knee with condyle height difference of 2.7 mm, and (c) knee with condyle height difference of 4.7 mm.

High knee flexion (>120°) requires significant translation of the femoral condyles on the tibial plateau. Inadequate femoral rollback and tibial rotation are common complications with current high-flexion prostheses during such high flexion [12–14]. To overcome these problems, Liu et al. developed a nonsymmetric CR tibial insert with a lateral condyle that was lowered and convex in shape, reportedly replicating the shape of a healthy knee [15, 16]. The convex insert allows the femoral condyle to sublux off the back of the tibial plateau and reduces the incidence of impingement during high flexion. Direct impingement between the posterior tibial insert and femur has been suggested as a factor limiting high flexion in conventional prostheses [17]. The concave radius of the medial condyle was also reduced to offer a tighter tibiofemoral contact. It was found that shaping the condyles of the CR TKR in this way increased femoral rollback and tibial internal rotation over a traditional symmetric TKR. However, it should be noted that beyond 100° knee flexion both the symmetric and nonsymmetric TKR models were reversed into external tibial rotation, but the intact (healthy) knee model continued to show internal rotation of the tibia [15]. So, while shaping the tibial insert similar to an intact knee does noticeably improve knee kinematics, it still cannot claim to accurately replicate the motion patterns of a healthy knee. A follow-up study modified the shape of both the medial and lateral condyles of the CR femoral component and included the aforementioned convex lateral tibial compartment (Figure 1) [18]. Rollback and rotation were compared to a symmetric TKR model. It was found that, by increasing the height of the medial femoral condyle over the lateral condyle, the model could demonstrate more natural knee motion from extension through to deep flexion, although this follow-up study did not directly compare the models against an intact knee. A general conclusion can be drawn that, by mimicking the convex shape of the lateral tibial insert and increasing the height of the medial femoral condyle over the lateral side, femoral rollback and tibial rotation can be improved.

Retrieval studies have consistently shown incongruent articular surfaces to be associated with a greater risk of polyethylene wear [19–21]. Increasing the conformity between the femoral and tibial surfaces and closely replicating the shape of the anatomical knee should help in reducing such wear. However, thinning the lateral compartment puts the insert at risk of fracture, and heightening the medial compartment may disrupt the joint line. Lin et al. [22] demonstrated that a 10 mm elevation of the joint line in a PS knee, by increasing the thickness of the tibial insert, significantly tensioned the collateral ligaments and increased joint stiffness. While stiffer ligaments may offer a more stable joint, such excessive stiffness as seen in Lin et al.'s study would place the joint under greater internal loading and possibly fracture the tibial insert. Further research is needed on this point to determine the optimal thickness of the insert and height difference between the medial and lateral compartments so as to offer the greatest biomechanical advantage without increasing the risk of component wear.

Additionally, mobile bearing tibial inserts have been developed to offer dual articulation in the knee, theoretically permitting a greater range of motion and reducing contact stress, and in turn reducing wear [19, 23]. In a series of case studies on retrieved implants, Huang et al. found such mobile bearings to produce smaller particulate debris and a higher percentage of granular debris in comparison to their fixed bearing counterparts, placing the knee at greater risk of osteolysis [24–26], but it was also noted that mobile bearing designs allow for earlier recognition of component wear [27].

**Figure 2:**
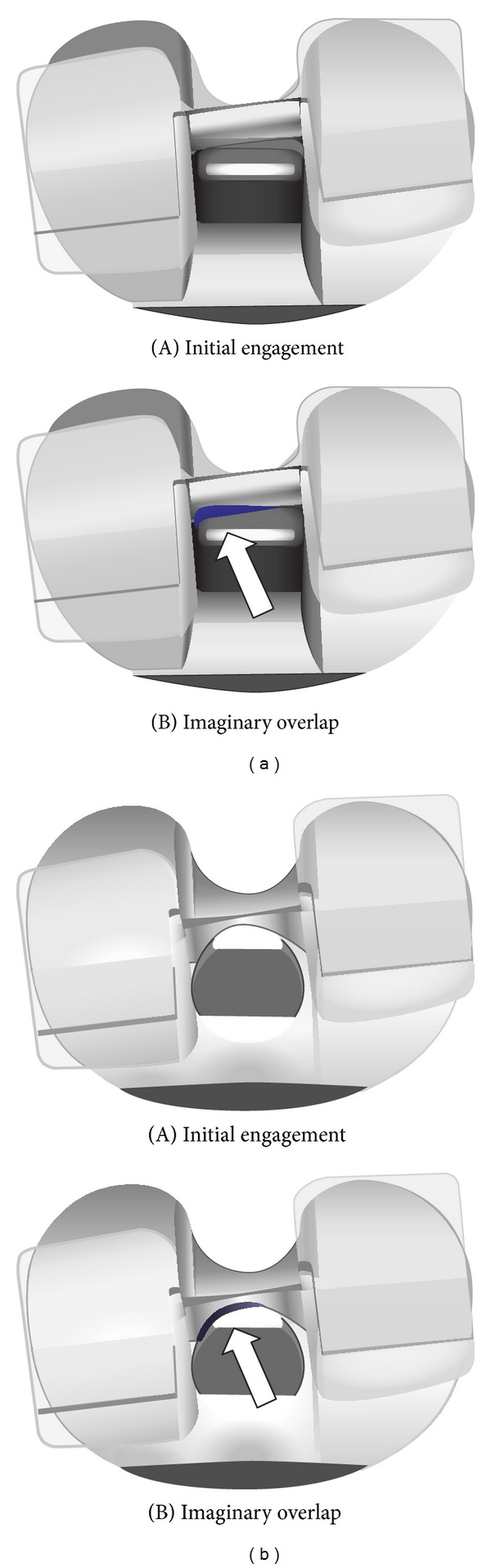
Initial engagement and the imaginary overlap between the tibial post and femoral cam for flat-on-flat (a) and curve-on-curve (b) models [28].

It has been reported that at least 19% of patients receiving posterior stabilized (PS) knees suffer abnormal tibiofemoral axial rotation [7]. While the degree of tibial rotation is highly variable between different TKA studies, it is generally conceded that axial rotation following TKA cannot accurately replicate healthy knee motion. Li et al. [14] demonstrated the importance of the cam-spine (post-cam) mechanism in guiding tibiofemoral motion and that knee motion after engagement between the post and cam was quite independent of the applied muscle loads. However, in PS knees, the interaction between the cam and spine as the knee is flexed, particularly at high flexion angles, will heavily influence both the motion of the knee and the longevity of the implant itself; a greater contact area will increase stability and reduce localized contact stress on the spine. Lin et al. evaluated two different post-cam contact shapes in PS knees, with flat-on-flat or curve-on-curve surfaces (Figure 2) [28]. Tibial rotation was shown to be comparable in both designs prior to post-cam engagement; an obvious deviation in plots was evident at around 45° knee flexion. The curve-on-curve design showed greater rotation beyond this point up to full flexion at 135°, although both designs followed a similar motion pattern. Figure 2 shows the overlap between the post and cam for both models during knee flexion. The greater medial impingement is obvious in the flat-on-flat model, which would increase edge loading on the post and also add resistance to tibial rotation.

**Figure 3:**
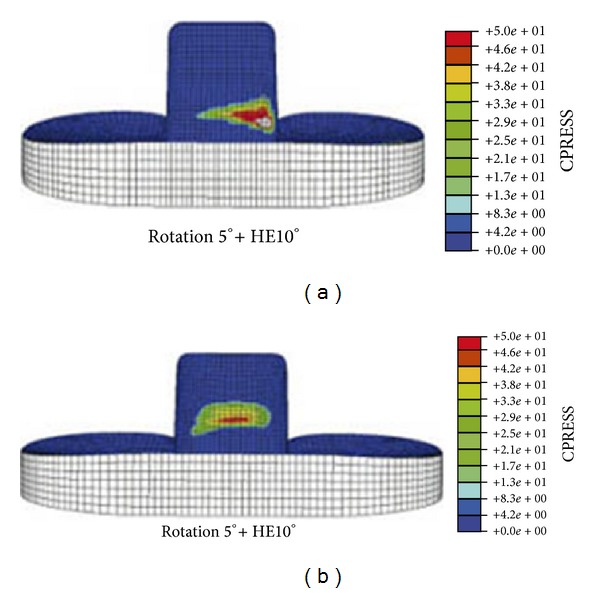
Contact stress (MPa) on the anterior face of tibial post at 10° hyperextension and 5° axial rotation for (a) flat-on-flat and (b) curve-on-curve contact surfaces (modified from [30]).

In a related study, Huang et al. analysed the stress on the tibial post for flat-on-flat and curve-on-curve designs up to a knee flexion angle of 150° [29]. Two conditions were simulated, one where there was no rotation between the tibia and femur and the other with 10° internal rotation of the tibial insert relative to the femoral component, which is more representative of anatomical alignment. Wear of the tibial post is inevitable as the cam and spine engage and rub against each other during knee flexion, and this can often lead to fracture of the post. As in Lin et al.'s study [28], a flat-on-flat design can be expected to experience greater edge loading on the posterior face of the post as the tibia rotates out of neutral alignment with the femur. For the 10° rotation models, Huang et al. showed that curve-on-curve contact surfaces can reduce the maximum contact stress on the posterior tibial post by over 30% in comparison to flat-on-flat surfaces and can increase the contact area by 8%. In a follow-up study, Huang et al. examined the contact status on the anterior face of the tibial post where contact with the femoral component occurs when the knee is extended [30]. Knee prostheses were modelled with 0°, 2.5°, and 5° of axial rotation and in 0°, 5°, and 10° of hyperextension; intuitively, hyperextension of the knee may further increase the stress on the anterior post. By tilting the femoral component forward by 5°, putting it into hyperextension, and tilting the tibial insert posteriorly by 5°, Wang et al. were able to improve femoral rollback in comparison to a knee with components inserted in a neutral alignment [31]. However, while the medial condyle showed comparable motion to a healthy knee up to 135° knee flexion, rollback of the lateral condyle was greatly reduced. Correspondingly, Huang et al. [30] showed that 10° of hyperextenion (with 5° axial rotation) could reduce anterior curve-on-curve peak contact stress by 25% in comparison to flat-on-flat surfaces and could increase the contact area by about 30% (Figure 3). Figure 3 details the location of the maximum contact stress when the knee was hyperextended by 10°; significant edge loading is obvious in the flat-on-flat model. Huang et al. also observed that the contact point shifted downward as the degree of hyperextension was increased from 0° to 5° to 10° and noted that the shorter moment arm could reduce the tensile stress on the post. Putting the knee into hyperextension may lower the contact point on the tibial post and improve femoral rollback, which is important for high flexion, but it also increases the stress on the anterior face of the post in comparison to a knee in neutral alignment.

In a bid to further improve tibial rotation, Lin et al. [32] modified a curve-on-curve surface to reduce the thickness of the medial side of the curved femoral cam; the cam gradually reduced in thickness from the lateral to medial side. In comparison to a baseline curve-on-curve model, the asymmetric cam design was shown to improve tibial rotation, but medial femoral rollback was compromised. Even after post-cam engagement, the medial condyle of the femur was located anteriorly, which could lead to early impingement.

## 4. Conclusion

In conclusion, these studies promote the use of anatomically shaped knee prostheses to achieve high knee flexion while limiting component wear. A rounded and convex lateral plateau and a shallower medial plateau on the tibial surface promote femoral rollback and permit more natural knee motion. Also, the post-cam contact surfaces should be rounded on both the anterior and posterior faces to reduce edge loading and produce a greater contact surface area throughout flexion. As mentioned, an asymmetric curve-on-curve cam shape may improve tibial rotation but at the expense of femoral rollback.
